# Sparse-View CT Joint Reconstruction Strategy with Sparse Sampling Encoding Layer

**DOI:** 10.2174/0115734056354972250221012822

**Published:** 2025-03-25

**Authors:** Hu Guo, Minghan Yang, Ziheng Zhang, Haibo Yu, Shuai Chen, Jianye Wang, Minghao Li

**Affiliations:** 1 University of Science and Technology of China, Hefei, 230026, Anhui, China; 2 Institute of Nuclear Energy Safety Technology, Chinese Academy of Sciences Hefei Institutes of Physical Science, Hefei, 230031, Anhui, China

**Keywords:** Imaging processing, Image reconstruction, CT, Sparse angular projection, Deep learning

## Abstract

**Background:**

Sparse angular projection is an important way to reduce CT dose. It consists of two processes, sparse sampling, and image reconstruction based on sparse projection. Under the traditional reconstruction framework, the sparseness of the projection angle may cause a degradation effect in the reconstructed image. A series of machine learning methods for sparse angle CT reconstruction developed in recent years, especially deep learning methods, can effectively improve the reconstruction quality, however, these methods can only reconstruct CT images based on a certain sparse sampling scheme.

**Objective:**

On the other words, they cannot search for an efficient sparse sampling scheme under a certain dose constraint automatically, which became the motivation to develop an end-to-end sparse angular CT reconstruction method.

**Methods:**

In this work, we propose a sampling encoding layer for searching sparse sampling schemes and integrate it into a sparse reconstruction neural network model based on projection data. Meanwhile, a joint reconstruction strategy based on both the radon domain and image domain painting is also developed.

**Results:**

Experiments based on public CT datasets demonstrate the effectiveness of the method.

**Conclusion:**

The results show that the joint reconstruction network based on a sparse sampling coding layer has great application potential.

## 1 INTRODUCTION

As a non-destructive testing method, computed tomography (CT) has been widely used in clinical diagnosis, security checks, and industrial testing. In the traditional CT reconstruction framework, dense projections in the range of or even more projected angles is usually required. However, complete dense projections may cause a problem with radiation dose [1], that is, the detected object has to absorb a high radiation dose, so this high-quality CT cannot be a detection method that can be used frequently [2].

Recently, low-dose CT (LDCT) has emerged as an alternative for rapid clinical evaluation and industrial testing because it has the safety-related advantage of reduced exposure to ionizing radiation [3]. LDCT can reduce the total dose based on two strategies [4]: (i) reducing the ray flux per projection (low flux projection), and (ii) reducing the number of projections (sparse-view projection). Since the ray flux can be adjusted by changing the scanning parameters (such as tube current, pitch, and tube potential/voltage), low flux projection has become a common LDCT strategy [5]. The degradation effect after LDCT reconstruction has also been widely studied as a special image denoise problem [6]. Given that the filtered back-projection (FBP) method is equipped on most commercial CT systems, the first strategy (low flux projection) may inevitably suffer degradation due to excessive noise and streak artifacts, often influenced by quantum noise and/or electronic noise [7]. Another strategy (sparse-view projection) to achieve higher reconstruction accuracy is iterative reconstruction. This encompasses two basic types: algebraic iterative reconstruction [8] and statistical iterative reconstruction [9]. While iterative reconstruction methods can significantly improve the reconstruction accuracy of LDCTs, they come with high computational complexity. This complexity can greatly reduce the efficiency of clinical examinations [7]. With the advancement of machine learning, especially deep learning, a large number of deep models for image denoising have played a pivotal role in LDCT reconstruction [10]. Examples include the wavelet domain residual network [11], the automated transform network based on AUTOMAP [12], the Generative Adversarial Network (GAN) [13] with Wasserstein distance and perceptual loss, and the Super-Resolution GAN [14] constrained by cycle learning ensemble, among others.

Sparse-view projection (including limited-view projection) provides the possibility of dose reduction from another perspective. Not only dose reduction, but also sparse-view projection has critical application requirements in some special medical and industrial applications, such as dental radiology, surgical imaging, mammography, and linear trajectory imaging systems. Sparse-view projection brings a matrix-solving problem under the condition of incomplete data, which is essentially different from the existence of low flux projection (the reconstruction based on low flux projection is essentially a denoising problem of projection). Over the past few decades, several researchers proposed some reconstruction methods based on sparse-view projection, such as reconstructing extended data using radon transform [15]; use prior information to reconstruct piecewise constant or otherwise sparse objects and reconstruct limited data with the algebraic and regularization methods. Similar to low flux projection reconstruction, some deep neural network models for sparse-view projection reconstruction were presented, for example, the automated transform network based on AUTOMAP presented by Zhu *et al*. [12]; the wavelet domain residual network developed by Zhang *et al*. [16]; the modular deep network based on encoder-decoder developed by Shan *et al*. [17].

The common feature of these deep network models with sparse-view projection matrix as input is that, they map sparse-view projections into a low-dimensional feature space based on a known sparse projection scheme, and then reconstruct the complete CT image from the low-dimensional feature [18]. While the design of a sparse projection scheme is hardly discussed in the machine learning framework. With the proposal of compressed sensing theory and its application in digital signal and image processing [19, 20], it can be known that the sparse representation and reconstruction of signals can be incorporated into an L_1_ optimization problem framework simultaneously. On this basis, some machine learning models for digital image compression were developed: Mousavi *et al*. presented a framework for sensing and recovering digital images based on stacked denoising autoencoder (SDA) [21]; Akshat *et al*. presented a compressive image recovery method based on the generative model [22]; Yao *et al*. designed a deep residual reconstruction network DR^2^-Net for image compressive sensing [23]; Kulkarni *et al*. designed a non-iterative reconstruction network of images ReconNet for compressively sensed measurements [24]. These researches have motivated us to build an end-to-end deep network for sparse-view projection CT reconstruction, that is, the end-to-end network that can not only reconstruct CT images based on sparse-view projection but also generate sparse projections.

In this work, we presented a projection-image joint reconstruction strategy with the sparse sampling encoding layer for sparse-view projection reconstruction. A sparse sampling layer can generate sparse sampling codes based on the complete projection scheme, where the activation function ReLU with sparse activation feature will play an important role. Based on the analysis of the degradation effect caused by sparse projection, we proposed a two-step strategy, that is, projection recovery based on the Radon domain first, and then detail reconstruction based on the image domain. Finally, the performance of the joint reconstruction strategy will be tested based on different datasets.

## 2 METHODS

To develop the reconstruction method, it is necessary to analyze the degradation process of CT images with sparse-view projection briefly. Then we will propose a joint reconstruction strategy with the sparse sampling network layer, and analyze the feasibility of the joint reconstruction strategy.

### 2.1 Degenerate Mechanism under Sparse-View Projection

The concise and intuitive nature of the photon counting model can help us understand key features of CT images with sparse-view projection [25]. On a given path of the photon through the imaged subject, it is assumed that 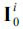 and **I***^i^* present the incident and the penetrated photon numbers respectively, and the subscript *i* presents the discrete index to the path of photon transmission, then the penetrated photon number of the path *i* can be given as an eq. (**1**):

**Table:** 

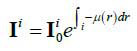	(1)

Where, *r* means the coordinate in the path *i* and *µ* is the attenuation coefficient when the photon acts. It is assumed that the energy distribution is ignored, and considering the projection noise, then the eq. (**1**) can be rewritten as follows:

**Table:** 

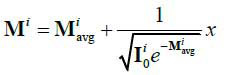	(2)

Where, 
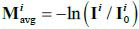
 represents the average degree of ray attenuation during CT. And *x* is a normally distributed random process with a mean of 0 and a variance of 1, *x* ~ N (0,1).

To analyze the effect of sparse sampling on CT imaging, the sparse sampling model should be established based on the eq. (**2**), which also means that we need to consider the response of **M***^i^* when **I***_0_* changes. If we ignore the low-dose degradation, **M***^i^* is constant (the degradation model in LDCT can be referred to [26], moreover, since the degradation term produced by a low dose has a superposition relationship with the original projection and the degradation term produced by sparse sampling mentioned below, they can be discussed independently). However, **I***_0_* in sparse-view projection is a function of the projection angle *θ*. To meet the needs of sparse sampling, the measurement of sparse-view projections 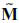 can be represented as follows eq. (**3**):

**Table:** 

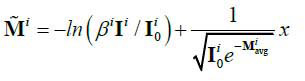	(3)

Where, *β^i^* is the sparse sampling coefficient relative to the original projection. Then if we consider the change in the projection angle *θ*, the measurement of sparse-view projections 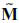 can be given as eq. (**4**):

**Table:** 

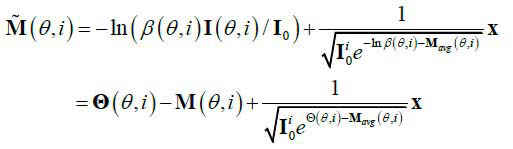	(4)

Where, 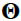 (*θ*,*i*)=-In *β*(*θ*,*i*). Then 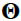 (*θ*,*i*) is the degradation item of projections under sparse-view projection. For facilitating the subsequent discussion, it can be set that 
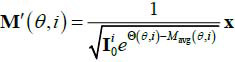
. Usually, most CT reconstruction is based on the FBP algorithm, so that the CT image can be represented as eq. (**5**):

**Table:** 

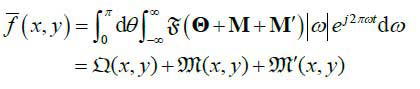	(5)

Where, F means Fourier transformation; Q,M and M' are integral transformations of 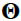 M and M'; and *t* = *x*cos*θ*+ *y*sin*θ*. Then, the reconstruction with sparse-view projections can be represented as the mapping G:

**Table:** 

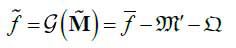	(6)

Eq. (**6**) shows that, the relationship between degradation items 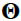, **M'** and **M** is superposition, so that the deep network with residual structure can be used for the feature extraction of **M'** and 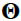, and reconstruction of 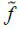 is similar to the LDCT reconstruction (that is, it only contains the degradation item **M'**).

### 2.2 Framework of Reconstruction Problem

Different from LDCT reconstruction, the sparse sampling scheme is required to be specifically formulated according to the features of the projection. If the total dose needs to be reduced to Γ*_l_* utilizing sparse sampling, then We can consider describing the reconstruction with sparse-view projections as a L_1_ norm optimization problem. It is assumed that **M***_j_* is introduced to represent the projection at an angle *θ* (eq. **7**):

**Table:** 

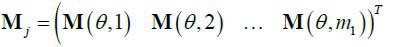	(7)

The original projection matrix consisting of projection **M***_j_* is **M***_x_* (**M***_x_* ϵM), and the orignal projection space M is shown as eq. (**8**).

**Table:** 

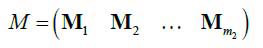	(8)

The size of M is *m*_1_×*m*_2_, which means *j* = 1,2,...*m*_2_, and the size of each projection **M**_*j*_ is a *m*_1_×1 vector. If the sparse projection matrix 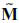 ϵ 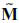
_Γ_l__, where, 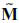
_Γ_l__ is the sparse projection space with the total dose Γ_l_, then the construction of the sparse projection scheme can be described as the mapping Π (eq. **9**):

**Table:** 

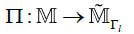	(9)


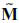
 can be further represented as eq. (**10**):

**Table:** 

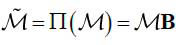	(10)

Where, 
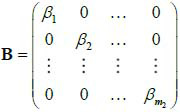
, which can be viewed as an encoding of the projection matrix, and the radiation dose of projection can be measured by the L_1_ norm 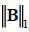.

In particular, for the original projections, the projection scheme can be described as the mapping Π_0 _ (eq. **11**):

**Table:** 

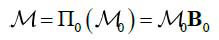	(11)

Where, if the projection matrix does not lose projections at any angles, then **B**_0 _ = **E**. The sparse projection scheme can be described as eq. (**12**) where G1 represents the projection encoding as shown in eq. (**12**):

**Table:** 

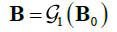	(12)

In fact, 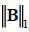 can be considered as the dose target. At the same time, we hope that the reconstruction model G2 can work as eq. (**13**):

**Table:** 

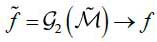	(13)

Then the reconstruction with sparse-view projections can be described as the L_1_ norm optimization problem (eq. **14**):

**Table:** 

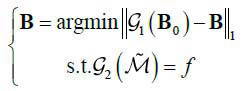	(14)

## 3 RECONSTRUCTION STRATEGY BASED ON JOINT DEEP MODEL

### 3.1 Basic Framework Projection Encoding and Image Reconstruction

In theory, the encoding model G_1_ and the reconstruction model G_2_ can be simultaneously built through machine learning. During the learning process, there is a training dataset 
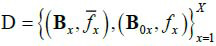
, where, the superscript *x* means the *x*th sample in the training set. The marginal distribution 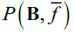 and the joint distribution 
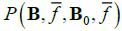
 can be approximated from **D**, so that the conditional probability 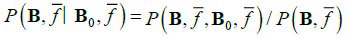 representing the CT reconstruction process with sparse-view projections can be obtained by a supervised learning directly.

**Table:** 

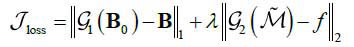	(15)

**Fig. (1):**
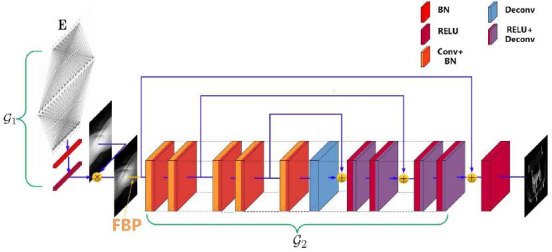
Encoding and CT image reconstruction model with 64 channels.

Due to the similarity of the reconstruction process, the supervised learning framework for low-flux may also be directly applied for sparse-view. According to the optimization problem 6, we can train the coupling model with encoding and reconstruction (Fig. **1**) based on the loss function (eq. **15**). In Fig. (**1**), a fully connected network (FCN) is used as the projection coding model G_1_, and a residual network consisting of convolution and deconvolution layers is used as the reconstruction model G_2_. In G_1_, we introduced a batch normalization (BN) layer to normalize each component of the FCN output and a special ReLU layer to process the normalized FCN output, to obtain sparse projection coding, where the ReLU function is given as eq. (**16**). In the ReLU function, we introduce a bias *λ'* to adjust the sparsity of projection coding. In particular, the residual network G_2_ has 5 Conv + ReLU and Deconv + ReLU layers with 64 channels respectively.

**Table:** 

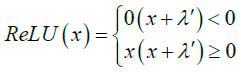	(16)

### 3.2 An Alternative within the Same Framework

**Fig. (2):**
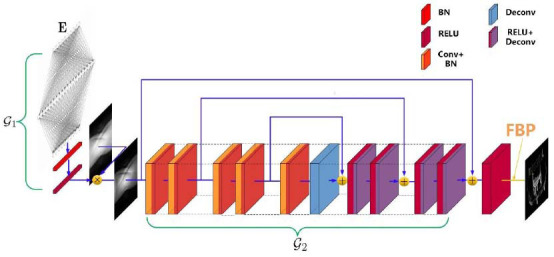
Encoding and CT projection reconstruction model 64 channels.

According to eq. (**2**), the information loss of the sparse-view projections is much greater than that of low-flux projections. Although both sparse-view and low-flux projections can be reconstructed with a similar learning framework, the reconstructed network of CTs with sparse-view projections may be more difficult to converge than that of CTs with low-flux projections. On the other hand, the deep network reconstruction model based on projection matrices (or sinograms) seems to be easier to learn. Sinograms do not contain complex spatial and density information, and we can infer the magnitude of missing projections from the adjacent projections. Then the complete-view projection matrix generated by the deep network can restore the general profile of the measured object by FBP. Then we can use the network structure similar to Fig. (**1**) to establish the sparse projection reconstruction model with sinograms as input (Fig. **2**).

However, the slight reconstruction error of the deep network in Fig. (**2**) may also be amplified by the integral transformation in the eq. (**5**). Therefore, a detailed reconstruction network based on CT image domain is also essential.

### 3.3 Joint Reconstruction

**Fig. (3):**
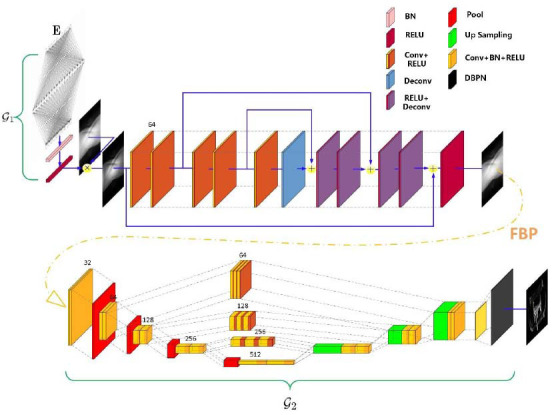
Encoding and joint reconstruction model.

Performance defects of the reconstructed network in Figs. (**1** and **2**) will be presented in section 4 through training process analysis and ablation experiments. To solve the above two problems, a joint reconstruction strategy was proposed. Firstly, the original projection M is recovered as much as possible according to the sparse projection 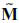, where, the projection reconstruction model shown in Fig. (**2**) is still used. Then the details of the projection reconstruction model output are further restored in the image domain by a similar approach to LDCTs reconstruction, where, we design a network with a structure similar to U-Net as the image detail restoration model (Fig. **3**).

The image detail restoration model has three parts. The first part is a low dimensional mapping, which can generate CT image profile features in a small scale. This model contains a convolution module and four 2× downsampling modules. The convolution module contains two Conv layers connected in series with the 3×3 kernel, which extracts the shallow features of CT images by increasing the width to 32 channels. Each downsampling module downsamples the module's input through the maxpool layer and utilizes two Conv layers connected in series to further double the width of the model. The second part is a super-resolution mapping that contains three 2× upsampling modules and one 2× super resolution module. The three upsampling modules consist of a linear interpolation layer and two Conv layers in series, and are connected with the super-resolution module through an additional convolutional layer with 1 channel. The construction of a super-resolution module is very flexible, any network with good 2× super-resolution performance can be considered. In this work, we adopt the Deep Back-Projection Networks (DBPN) with 1 channel as the super-resolution module. The three-part is three skip connections, where each skip connection contains three Conv+ReLU layers in series. These skip connections strike a balance between obtaining detailed information and eliminating degradation effects. In this way, the image reconstruction model and the projection reconstruction model are coupled together through FBP to form the joint reconstruction model proposed in this study.

Accordingly, the training process based on loss function (15) need to be modified to accommodate the joint reconstruction strategy. A three-steps training process is adopted:

1. Step 1: According to the dose target 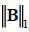, set the value of the bias *λ'*;

2. Step 2: Take the complete CT projection matrix M and projection encoding **E** as the inputs, training the subnetwork G_1_ ◦ G'_2_ based on the loss function eq. (**17**):

**Table:** 

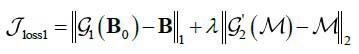	(17)

3. Step 3: Take the output 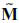 of the sub-network G_1_ ◦ G'_2_ as the input of the sub-network G"_2_, training the sub-network G_1_ ◦ G'_2_ based on the loss function eq. (**18**).

**Table:** 

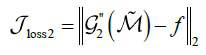	(18)

## 4 RESULTS AND DISCUSSION

In this section, we evaluated the performance of the joint reconstruction strategy for sparse-view CT from three aspects. Firstly, we showed the training process, results, and image quality evaluation metrics (including the mean square error (MSE), peak signal-to-noise ratio (PSNR), and structural similarity index measure (SSIM)) of the joint reconstruction model; then we analyzed the performance of the sparse-view projections generated by G_1_; Last, at a specific dose limit, we compared the joint reconstruction strategy based on G_1_ with the models in Figs. (**1** and **2**), traditional frequency domain denoising methods, and typical deep learning methods.

### 4.1 Dataset

3 datasets were used in this section for performance evaluation of the joint reconstruction strategy with sparse-view projection encoding:

1. Thoracic phantoms in the FDA dataset: The 512×512 NDCTs in this data set come from Phantom FDA, Cancer Imaging Archive, and we generated the corresponding LDCTs with 20%, 30% and 40% of routine dose with the same method as the chest dataset on this. The 104 scanned objects of this dataset are the anthropomorphic thoracic phantom 105 (Kyotokagaku Incorporated, Tokyo, Japan) rather than clinical patients. 106 These phantoms were scanned by a Philips 16-row scanner (Mx8000 IDT, 107 Philips Healthcare, Andover, MA) and a Siemens 64-row scanner (Somatom 108 Definition 64, Siemens Medical Solutions USA, Inc., Malvern, PA) [27].

2. Multicenter CT phantoms public dataset: This dataset is acquired at three centers and collected especially for radiomics reproducibility research. Three phantoms were scanned in three independent institutions. Images of the following phantoms were acquired: Catphan 700 and COPDGene Phantom II (Phantom Laboratory, Greenwich, NY, USA), and the Triple modality 3D Abdominal Phantom (CIRS, Norfolk, VA, USA). Data were collected at three Dutch medical centers: MAASTRO Clinic (Maastricht, NL), Radboud University Medical Center (Nijmegen, NL), and University Medical Center Groningen (Groningen, NL) with scanners from two different manufacturers Siemens Healthcare and Philips Healthcare [28].

3. The American Association of Physicists in Medicine (AAPM) Low-Dose X-Ray CT Grand Challenge [29]: The patient data library consists of contrast-enhanced abdominal CT examinations selected by the host institution. All data were obtained on similar scanner models (Somatom Definition AS+, or Somatom Definition Flash operated in single-source mode, Siemens Healthcare, Forchheim, Germany). CT image data reconstructed using the commercial CT system are provided for the full dose projection data. All CT images were reconstructed using a filtered back projection method. In this work, we choose 10 cases of full-dose chest CTs to build a new dataset. The original dataset contains projection data and reconstructed CT images.

### 4.2 Parameter Selection

In experiments of this work, we evaluated several parameter combinations and finalized the parameter settings as follows:

1. Learning rate: The base learning rate was set to 1×10^-5^.

2. Kernel size: The kernel size of all layers was set to 3×3.

3. Stride: The strides of convolution and deconvolution were set to 1 with no padding.

4. Dose target: For evaluating the network performance, we introduce three dose values: 40%,30%,20%.

5. experimental environment: All experiments were performed with Python 3.6, PyTorch 1.6.0, and CUDA 10.1 on computer workstations of Hefei Advanced Computing Center.

### 4.3 Analysis of Training Process

#### 4.3.1 Convergence of Training Process

**Fig. (4):**
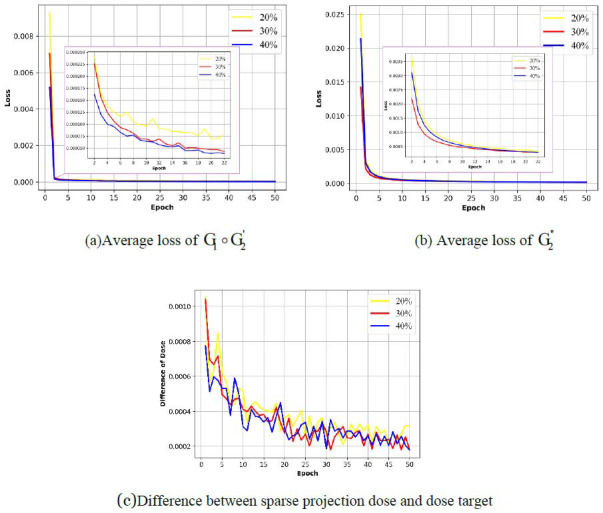
(**a**): Average, (**b**): Loss and (**c**): Dose difference of each epoch during training process (Multicenter CT phantom dataset).

To illustrate the convergence of the joint reconstruction strategy, we plotted the average loss (Fig. **4**) and the difference between the sparse projection doses and dose targets in each epoch during the training process (Fig. **4**). In each figure, we plotted the curves under three different dose targets: 40%,30% and 20%. In the initial stages of training processes of the sub-network G_1_ ◦ G'_2_ and G"_2_ (1-10 epochs in (Fig. **4a** and **b**), the loss under high dose (40%) is significantly lower than the loss under low doses (30% and 20%). This shows the difference in the reconstruction process under different sparse degrees, that is, the lower the target dose, the sparser the projection, and then the more difficult it is to search the projection encoding that meets the constraints in the feasible domain in the eq. **14** and reconstruct the projection matrix M. Finally, the training process of G_1_ ◦ G'_2_ and G"_2_ converged after 20 and 15 epochs respectively, which means feasible solutions satisfying different dose constraints can be found. We also calculated the normalized dose differences between the sparse projection doses and dose targets 40%,30% and 20% in each training epoch. Similarly, the dose difference under 40% converges faster than the dose difference under 30% and 20%.

#### 4.3.2 Quality Evaluation

**Fig. (6):**
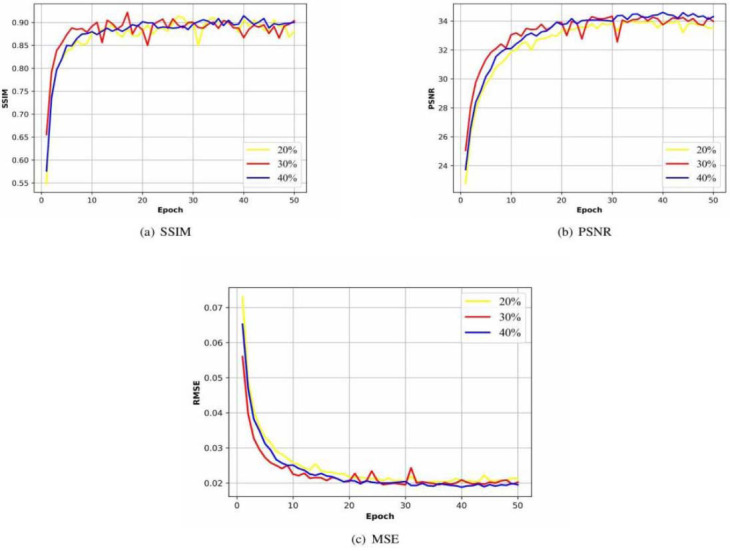
(**a**) SSIM, (**b**) PSNR and (**c**) MSE of sub-network G_2_" (Multicenter CT phantoms dataset).

After each training epoch, we evaluate the image quality based on the image quality metrics (SSIM, PSNR and MSE, shown in Figs. (**5** and **6**), which also shows a convergence trend similar to the loss function in (Fig. **4a** and **b**). Moreover, they also reflect the further repairing effect of G_2_'" on G_2_'. The SSIM, PSNR and MSE in Fig. (**5**) shows the high similarity between the reconstructed projection matrix and the original projection matrix after step 2. While in the initial phase of step 3 (Fig. **6**), the image quality metrics remain at low levels until after about 20 epochs, the image quality metrics gradually converge.

#### 4.3.3 Visualization of Sparse Projection Encoding and Reconstruction Image

We extracted the intermediate results of the encoding and joint reconstruction model in Fig. (**3**) to intuitively show the role of the encoding sub-network and joint reconstruction sub-network. First, we will visually show the sparse coding and the corresponding sparse projection matrix. Under the combined action of the FCN, the BN layer, and the ReLU layer, the projection code of G_1_ output has sparse characteristics, and the required dose of each angle is different. Then, the sub-network G'_2_ reconstructed the complete projection based on the sparse-view projection, while compared with the original projection, the reconstructed projection is still different in many details, which reflects that it is not enough to rely on projection repair alone. Sub-network G"_2_ repairs the details in the image domain, which further reduces the difference between the reconstructed image and the original image. This also matches the performance changes shown in Fig. (**6**).

### 4.4 Ablation Experiment

#### 4.4.1 Joint Reconstruction

In this section, we will first take the FDA dataset and 40% target dose as the example, and conduct two ablation experiments to demonstrate the necessary for joint reconstruction: reconstruction sub-networks G'_2_ and G"_2_. For this purpose, we constructed the other two encoding and reconstruction network G_1_ ◦ G'_2_ and G_1_ ◦ G"_2_ besides the original network G_1_ ◦ G'_2_ ◦ G"_2_. The image quality metrics of the three networks during the training process are given as Fig. (**7**), where, quality metric curves of the complete network are the same as curves in Fig. (**6**). After 50 epochs, the SSIM. PSNR and MSE of G_1_ ◦ G'_2_ and G_1_ ◦ G"_2_ are significantly lower than the original complete network. As mentioned in section 3, the integral transformation can magnify errors in projection matrices, so that metrics of G_1_ ◦ G'_2_ are always at lower levels (after 50 epochs, the SSIM, PSNR, and MSE can only get to 0.56). On the other hand, the metrics of the network G_1_ ◦ G"_2_ can converge quickly, but it is also significantly lower than the metrics of the complete network. It shows that the direct sparse reconstruction in CT image domain is very difficult.

#### 4.4.2 Encoding

The effect of sparse projection encoding generated by the encoding network on reconstruction can also be demonstrated by the ablation experiment. For comparison, we took the 40% target dose as the example, and introduced an equal interval sampling scheme, which is the common sampling scheme. As shown in Fig. (**8**), after training with 20 epochs in Step 2, we can find the difference between the two schemes significantly. In the training process, the loss of the two schemes converges rapidly. However, unlike the SSIM curve in Fig. (**5a**), the SSIM of the reconstruction model with the equal interval scheme does not increase significantly (changing around 0.5), and even decreases. It demonstrates the effectiveness of the sparse reconstruction sub-network G_1_, that is, under a determined dose constraint, the sparse projection matrix obtained without the optimization process as shown in eq. (**14**) may not be repaired by the joint reconstruction sub-network G'_2_ ◦ G"_2_.

### 4.5 Comparison Experiment

Under the same task objectives (sparse-view projection CT reconstruction), we compared the performance of the proposed sparse encoding and joint-reconstruction network (SEJR), traditional reconstruction method (FBP and algebraic reconstruction technique, ART), and the other state-of-the-art network (DnCNN, U-Net, and RED-CNN). For the proposed method in this work, all the other methods take equal interval sampling projection matrices as the input. Since it is shown in section 4.4.2 that it is very difficult to repair equal interval sampling matrices directly, they will be processed by FBP to improve the probability of reconstruction.

The comparison experiment was conducted based on the FDA dataset and AAPM dataset, and the target dose was set at 20% on FDA dataset and 50% on AAPM dataset. Image quality metrics of the proposed sparse encoding joint reconstruction model and other methods to be compared are shown as Tables **1** and **2**. Compared with the traditional reconstruction methods (FBP and ART) and deep networks CNN10 and RED-CNN, SEJR has large performance advantages on all metrics; meanwhile, compared with the U-Net, which has a reconstruction process similar to SEJR in the CT image domain, the SEJR still has higher image quality evaluation metrics. These differences of image quality evaluation metrics show SEJR's advantages in sparse projection scheme and joint reconstruction: firstly, for deep networks other than SEJR, their sparse projection scheme is not optimal; then, the ability of these deep networks to reconstruct images only in the image domain is limited.

#### 4.5.1 Phantoms Experiment on FDA Dataset

Taking one CT image in the FDA dataset as an example, the visual differences between CT images reconstructed by SEJR and other methods are shown in Fig. (**9**), where the first is the normal CT image as the gold standard. Two regions of interest (ROIs) were selected for detailed comparison (ribs shown in the yellow boxes, and thoracic vertebras shown in pink boxes). The ROI presented by SEJR is almost the same as in the normal CT image, with only a small amount of reconstruction error around the ribs. The ROIs output by the U-Net not only have striped artifacts but also are blurrier than the normal CT image. Outputs of the CNN10 and REDCNN not only have striped artifacts but also some unexpected ring artifacts. Compared to the SEJR and U-Net, CNN10, and RED-CNN have no improved more significantly than traditional FBP and ART, which is consistent with the quality evaluation metrics shown in Table **1**.

#### 4.5.2 Medical Experiment on the AAPM Dataset

Then we conduct a comparative experiment of different sparse strategies on the AAPM dataset. Taking one CT image with 50%, 40%, and 30% target dose in the AAPM dataset as an example, the visual differences between CT images reconstructed by SEJR and other methods were shown in Fig. (**10**). The edge of the image is cut slightly, and irrelevant information about the edge is ignored. Two regions of interest (ROIs) were selected for detailed comparison. The results performance trend on the medical dataset is similar to the representation trend on the phantom dataset. The ROI presented by SEJR is almost the same as in the normal CT image, with only a small difference but does not affect the texture of the structure. U-Net has less texture deformation, but the edges of some structures are not clear enough. The reconstruction results of REDCNN, FBP, and ART all have serious artifacts, and the disadvantages of sparse projection are not well dealt with. The ROIs output by the U-Net not only have striped artifacts but also are blurrier than the normal CT image. Outputs of the CNN10 and REDCNN not only have striped artifacts but also some unexpected ring artifacts. Compared to FBP, ART, and REDCNN, CNN10 has improvements in artifacts, but the overall details are fuzzy, which is consistent with the quality evaluation metrics shown in Table **2**.

**Fig. (10a-c):**
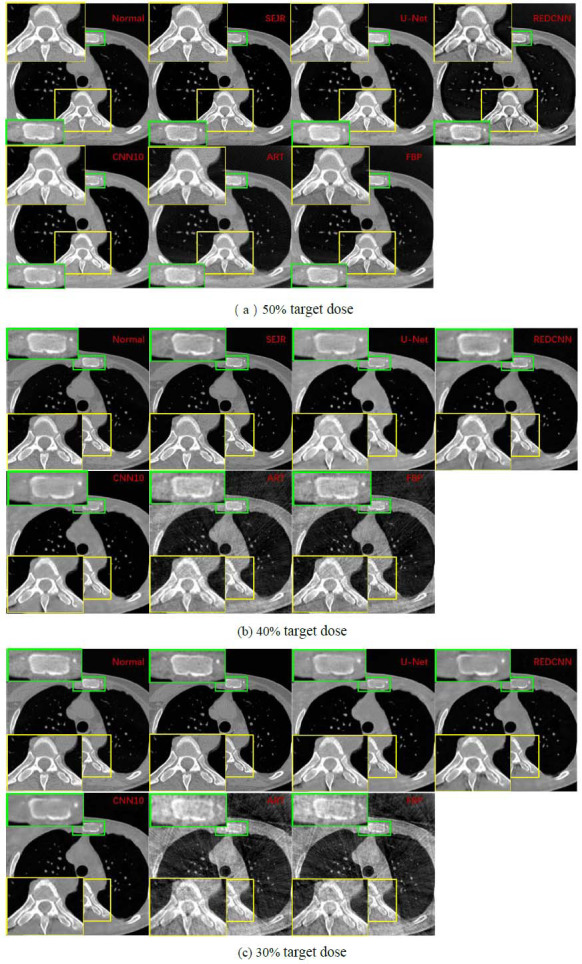
Two regions of interest (ROIs) from the visual differences of the different target dose CT image in the AAPM dataset reconstructed by SEJR and other methods were selected for detailed comparison.

We also found something interesting in the direction of sparsity. As the CT images become sparser, different methods exhibit varying performances. From Fig. (**10**) , it can be seen that the linear change in sparsity level does not linearly affect the visual effects of FBP and ART, and the impact is significant, but the impact on several deep learning methods is not very large. Among them, the U-Net, REDCNN, and CNN10 structures exhibit varying degrees of artifacts at the edges. The visual effects of SEJR have consistently remained excellent, but when the sparsity level is further reduced, there is a slight blurring in the SEJR structure, which should be the main reason for the decrease in its metrics. As can be seen from Table **2** , all metrics of REDCNN have improved to varying degrees at 40% sparsity, especially SSIM. In combination with (Fig. **10**) , it can be seen that artifacts of the edge structure are eliminated to a certain extent. At a sparsity level of 30%, the edge artifacts of REDCNN are enhanced again, leading to a decrease in the metrics PSNR and MSE. Surprisingly, SEJR still maintains a leading position in terms of visual effects and metrics in all comparisons. The reconstruction results of the other methods are consistent with (Fig. **10**) , showing a significant decrease in performance in all aspects.

## CONCLUSION

In this work, a complete method for sparse projection and CT image reconstruction has been proposed. We first presented a sparse projection automatic search scheme for CT image based on the sparse sampling encoding layer; second, we constructed a joint reconstruction strategy in the projection domain and image domain respectively; then we coupled the sparse sampling encoding layer with the joint reconstruction network, and optimize the network parameters through the joint loss formed by the projection dose 
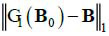
 and the reconstruction error 
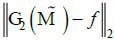
 of CT images. The thoracic phantoms in the FDA dataset and multicenter CT phantoms public dataset were utilized to evaluate and validate the performance of our proposed method in comparison with traditional and typical deep learning methods. The results showed a great potential of the joint reconstruction network with the sparse sampling encoding layer. For the imaging task of a specific object (such as the chest, liver, or mechanical part), constructing a sparse projection scheme through the sparse sampling encoding layer will help to reduce the number of projections and also be able to improve the CT reconstruction accuracy. In addition, the proposed joint reconstruction of CT images with sparse projections in the projection domain and the image domain has a stronger performance than the reconstruction scheme only in the image domain. In the future, we plan to carry out sparse-view CT reconstruction experiments based on the projection scheme given by the sparse sampling encoding layer on a real CT device.

## Figures and Tables

**Fig. (5):**
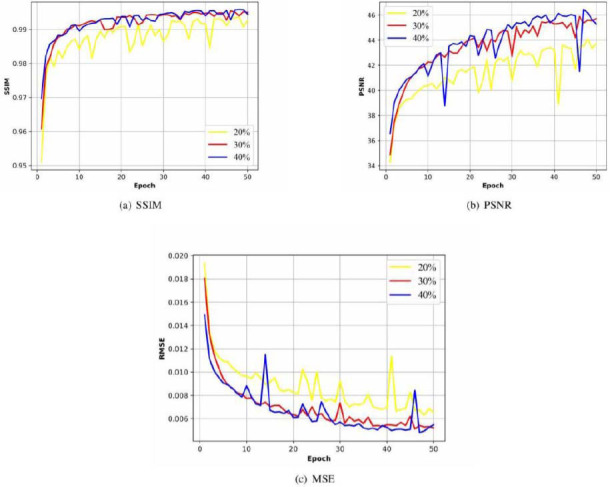
(**a**) SSIM, (**b**) PSNR and (**c**) MSE of sub-network G_1_ ◦ G_2_' (Multicenter CT phantoms dataset).

**Fig. (7):**
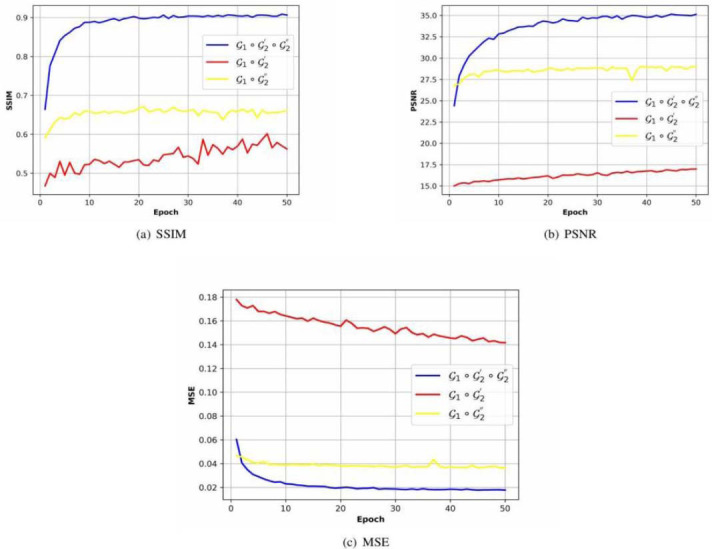
(**a**) SSIM, (**b**) PSNR and (**c**) MSE Comparison among the three networks (40% target dose): (**a**) complete sparse encoding and joint reconstruction network G_1_ ◦ G'_2_ ◦ G"_2_; (**b**) sparse encoding and projection reconstruction network G_1_ ◦ G'_2_; (c) sparse encoding and CT image reconstruction network G_1_ ◦ G"_2_.

**Fig. (8):**
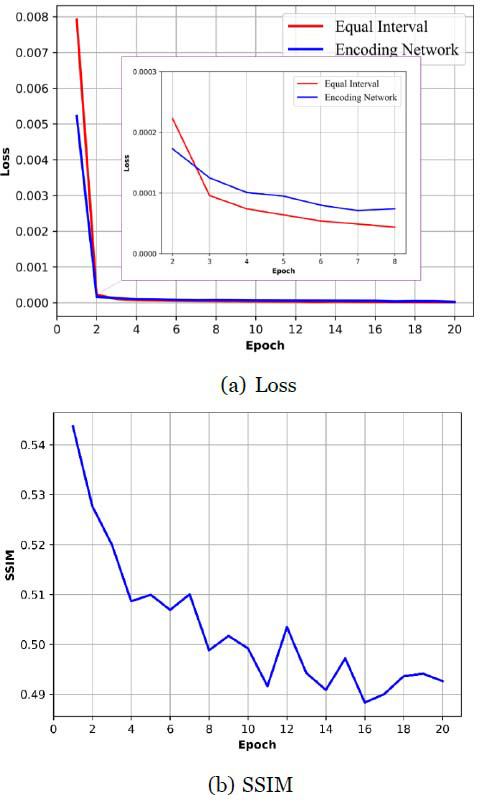
Performance comparison between the reconstruction network with sparse encoding sub-network and equal interval sampling scheme (40% target dose): (**a**) Loss function in the training process of the Step 2; (**b**) SSIM after each epoch in the training process of the Step 2.

**Fig. (9):**
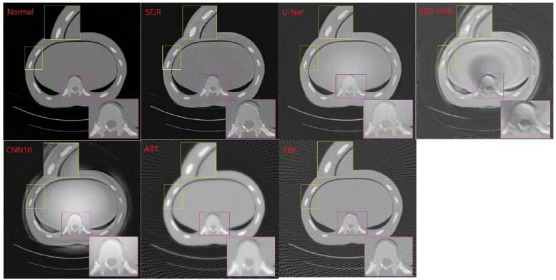
Two regions of interest (ROIs) from the visual differences of the 20% target dose CT image in the FDA dataset reconstructed by SEJR and other methods were selected for detailed comparison.

**Table 1:** Image quality evaluation metrics (mean ± SDs) associated with different methods (FDA dataset, 20% target dose).

**-**	**SSIM**	**MSE**	**PNSR**
SEJR	0.8906±0.0078	0.0212±0.0033	33.5274±1.3411
CNN10	0.5758±0.0278	0.2446±0.0064	12.2521±1.8147
RED-CNN	0.6923±0.0308	0.0741±0.0203	22.9155±2.2778
U-Net	0.8793±0.0089	0.0280±0.0052	31.4052±1.5625
FBP	0.5728±0.0088	0.0452±0.0027	13.4452±0.2798
ART	0.6606±0.0106	0.0427±0.0027	13.7033±0.2747

**Table 2:** Image quality evaluation metrics (mean ± SDs) associated with different methods (AAPM dataset, different target dose).

** Target Dose **	** Methods **	** SSIM **	** MSE **	** PNSR **
50%	SEJR	0.8550±0.0356	0.0278±0.0036	31.0659±1.1786
	CNN10	0.7706±0.0309	0.0878±0.0126	21.1623±1.6823
	RED-CNN	0.6851±0.0863	0.0949±0.0692	20.5104±1.9341
	U-Net	0.7759±0.0452	0.0652±0.0133	24.09341.2132
	FBP	0.6244±0.0167	0.1324±0.0253	17.6136±0.3929
	ART	0.6992±0.0292	0.1118±0.0167	19.0805±0.6833
40%	SEJR	0.8096±0.0818	0.0395±0.0038	28.0590±0.8717
	CNN10	0.7307±0.0509	0.0880±0.0122	21.1497±1.7800
	RED-CNN	0.7079±0.0284	0.0535±0.0124	25.6546±2.0175
	U-Net	0.6008±0.0722	0.1202±0.0200	18.5183±1.4228
	FBP	0.4006±0.0293	0.1132±0.0111	18.6939±0.8782
	ART	0.4977±0.0219	0.2306±0.0921	17.9020±2.0467
30%	SEJR	0.7434±0.0657	0.0369±0.0030	28.3214±0.7557
	CNN10	0.6177±0.0679	0.1186±0.0139	18.5713±1.0187
	RED-CNN	0.7223±0.0319	0.0645±0.0151	24.0564±2.1375
	U-Net	0.5956±0.0723	0.1230±0.0211	18.3305±1.5178
	FBP	0.3252±0.0246	0.1561±0.0142	15.9227±0.8082
	ART	0.4537±0.0168	0.2417±0.0854	17.4889±2.5954

## Data Availability

All the data and supporting information is provided within the article.

## LIST OF ABBREVIATIONS

CT: Computed tomography
LDCT: Low-dose CT
FBP: Filtered back-projection
GAN: Generative Adversarial Network
SDA: Stacked denoising autoencoder
BN: Batch normalization
DBPN: Deep Back-Projection Networks
MSE: Mean square error
SSIM: Structural similarity index measure
PSNR: Peak signal-to-noise ratio
ROIs: Regions of interest
